# Understanding the relationship between surfing performance and fin design

**DOI:** 10.1038/s41598-024-58387-y

**Published:** 2024-04-16

**Authors:** James R. Forsyth, Grant Barnsley, Mehrdad Amirghasemi, Johan Barthelemy, Alhoush Elshahomi, Buyung Kosasih, Pascal Perez, Stephen Beirne, Julie R. Steele, Marc in het Panhuis

**Affiliations:** 1 Biomechanics Research Laboratory, University of Wollongong, Wollongong, NSW 2522 Australia; 2 Australian Institute for Innovative Materials, University of Wollongong, Wollongong, 2522 Australia; 3 SMART Infrastructure Facility, University of Wollongong, Wollongong, 2522 Australia; 4 School of Mechanical, Materials, Mechatronic and Biomedical Engineering, University of Wollongong, Wollongong, NSW 2522 Australia; 5 Surf Flex Lab, University of Wollongong, Wollongong, NSW 2522 Australia; 6 School of Chemistry and Molecular Bioscience, University of Wollongong, Wollongong, NSW 2522 Australia

**Keywords:** Engineering, Translational research

## Abstract

This research aimed to determine whether accomplished surfers could accurately perceive how changes to surfboard fin design affected their surfing performance. Four different surfboard fins, including conventional, single-grooved, and double-grooved fins, were developed using computer-aided design combined with additive manufacturing (3D printing). We systematically installed these 3D-printed fins into instrumented surfboards, which six accomplished surfers rode on waves in the ocean in a random order while blinded to the fin condition. We quantified the surfers’ wave-riding performance during each surfing bout using a sport-specific tracking device embedded in each instrumented surfboard. After each fin condition, the surfers rated their perceptions of the Drive, Feel, Hold, Speed, Stiffness, and Turnability they experienced while performing turns using a visual analogue scale. Relationships between the surfer’s perceptions of the fins and their surfing performance data collected from the tracking devices were then examined. The results revealed that participants preferred the single-grooved fins for Speed and Feel, followed by double-grooved fins, commercially available fins, and conventional fins without grooves. Crucially, the surfers’ perceptions of their performance matched the objective data from the embedded sensors. Our findings demonstrate that accomplished surfers can perceive how changes to surfboard fins influence their surfing performance.

## Introduction

Surfing is rapidly becoming a global sport following its inclusion in the Tokyo 2020 Olympic Games. Currently, the surfing equipment market is valued at approximately US$4 billion worldwide, with the North American market holding nearly 50% of the market share^1^. Although the primary piece of surfing equipment is a surfboard, the type of fin selected and fitted to a surfboard directly influences the performance of a surfer’s board.

The fins of a surfboard interact with the water flow around it to give the surfer control and maneuverability^2^. Previous research has shown that the design of a fin (construction, shape, surface features)^2–4^ and the arrangement or number of fins^5–7^ can influence the flow of water around the fin. Based on fluid dynamics, changes to water flowing around a fin will change the lift and drag forces acting on the fin and, in turn, how the surfboard performs. Surfers can, therefore, select the type of fin that best suits their aesthetic, functional, or performance goals when surfing from the vast array of fins marketed by surfboard fin manufacturers. However, limited research has investigated the human-equipment interaction to evaluate whether surfers can perceive how different fin arrangements, shapes, and constructions affect their performance.

Previous studies^3,4^ have provided some of the first performance data on surfboard fins, monitoring the movement of surfers’ boards with a commercial, sport-specific device containing nine inertial sensors and a Global Positioning System (GPS). These data were used to evaluate the functional performance of fin construction^3^ and surface features^4^, using outcome measures such as speed, turn angle, turn duration and more. Notably, by changing the surface features so that the fin represented that of a Humpback Whale’s pectoral fin, surfers displayed significant increases in performance outcomes such as cutback yaw power and power/inertia^4^. Power/inertia (P/I), calculated using the difference in angular velocity between two characteristic surfing maneuvers (bottom turn and cutback)^3,4^, was proposed as a measure of total turning power because it eliminated the effects of inertia. Although these findings provide interesting insights into how fins can influence surfing performance, these results were limited to only a few participants surfing in varied conditions.

In the current surfing equipment market, most design decisions are based on subjective assessments or perceptions of a piece of equipment’s performance. Very little published evidence informs the design features and characteristics of surfing equipment^8^. However, previous studies have examined the perception of “feel” or performance of equipment in other sports such as golf^9^, skiing^10^, and tennis^11^. In these studies, the researchers noted that experienced sports people could perceive the mechanical or performance qualities of the equipment they used when components were modified. As the mechanical properties of sporting equipment can influence performance, it is imperative that athletes can identify equipment that is likely to enhance their functional performance. In surfing, we recently reported that the lift-to-drag ratio of surfing fins can be improved by 11% by adding a grooved outer edge to a commercial fin template^2^. However, this improvement was based on theoretical modelling using computational fluid dynamics rather than quantifying changes in the performance of surfers riding waves. As such, it is unclear whether surfers can perceive any benefits of changes to fin design when they surf.

The purpose of this study was twofold: (1) to determine whether surfboard fins of varied material composition and design could influence the performance of surfers riding waves in the ocean, and (2) to determine whether accomplished surfers could perceive how changes to surfboard fin design affected their performance while surfing waves in the ocean. We hypothesized that adding surface features to fins would improve the performance of the surfers when riding waves in the ocean. Additionally, we believed that accomplished surfers would be able to perceive changes in their surfing performance caused by different fin designs.

## Results

### Fin design and surfing performance

**Figure 1:**
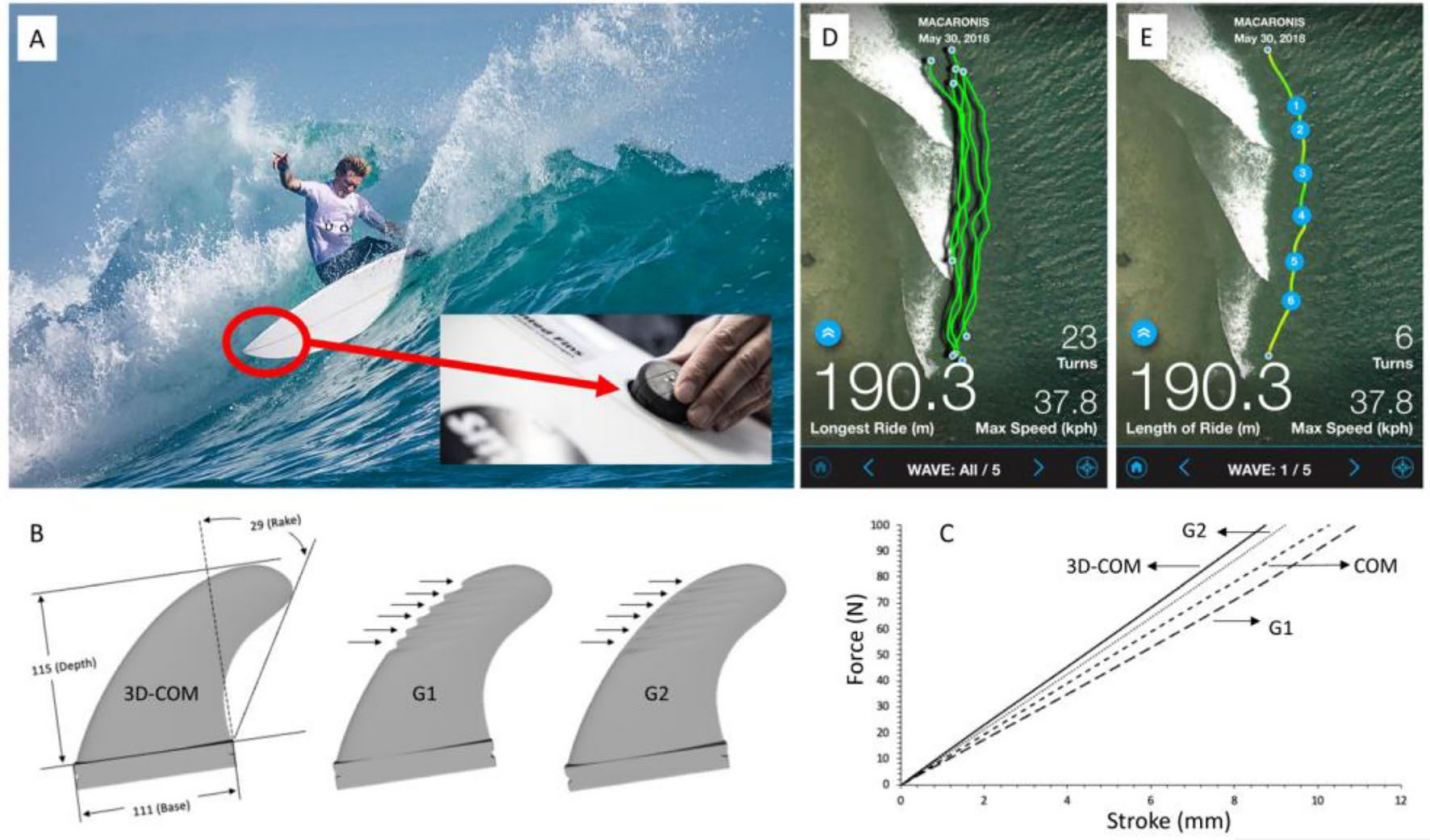
A composite image of (**A**) A participant performing a cutback with the experimental fins during testing with the location of the sport-specific tracking device circled. Inset: Image showing the sport-specific tracking device being embedded flush with the surfboard deck. (**B**) CAD designs for the 3D-COM, G1, and G2 fins are shown with a single-tab base. Dimensions of the 3D-COM fin are shown in millimeters (for depth and base) and degrees (for rake). The arrows on the G1 and G2 fins indicate the grooves’ position on each fin's outer surface, which were 60 mm long and separated by 6 mm. All fins are shown with single-tab bases. (**C**) Applied force as a function of applied stroke for typical fin testing of the commercial and 3D-printed fin designs with single-tab base. (**D**) Image showing Global Positioning System (GPS) data for five waves during a typical testing session. (**E**) Image showing GPS data for the first wave during the testing session shown in (**D**). Numbers 1–6 indicate the GPS location of measured bottom turns/cutbacks.

Four fin designs (on both single- and double-tab fin bases) were tested in this study—(1) a commercial fin (COM), (2) a 3D printed fin with the same shape and dimensions as the commercial fin (3DCOM), (3) a 3D printed fin with a grooved outer surface (G1), and (4) a 3D printed fin with a grooved outer and inner surface (G2; see Fig. 1). That is, the primary differences between the fin conditions were in the method of construction (i.e., injection moulding vs 3D printing) and fin design (i.e., no surface features vs grooved surfaces). We performed a series of tests to characterize the mechanical differences in stiffness and flex pattern among the four fin conditions (see Table 1). Lower stiffness values were found for the single tab and double tab fin base in the fins with added surface features.

**Table 1:** An overview of the structural and functional differences of the four fin conditions, including means ± 95% confidence intervals (CI; where relevant).

Fin condition	Fin base type	Materials/design features	Modulus (N/mm)
COM	FCS	Fiberglass + honeycomb matrix	9.7 ± 0.1
	Futures	Fiberglass + honeycomb matrix	11.0 ± 0.1
3DCOM	FCS	Nylon carbon composite + continuous carbon fiber	10.7 ± 0.1
	Futures	Nylon carbon composite + continuous carbon fiber	11.7 ± 0.1
G1	FCS	Nylon carbon composite + continuous carbon fiber	8.0 ± 0.1
	Futures	Nylon carbon composite + continuous carbon fiber	10.1 ± 0.1
G2	FCS	Nylon carbon composite + continuous carbon fiber	8.3 ± 0.1
	Futures	Nylon carbon composite + continuous carbon fiber	10.5 ± 0.1

COM refers to commercially available fins produced via injection molding. 3DCOM, G1, and G2 indicate fins produced by additive manufacturing (3D printing) using a continuous fiber reinforcement method.

Across 6 days, six surfers (mean age: 31 ± 6 years, height: 180 ± 9 cm, mass: 76 ± 11 kg, experience: 22 ± 5 years) participated in six surfing sessions at one surfing location (Macaronis, Mentawai Islands, Indonesia). The mean wave power over the surfing sessions was 450 ± 30 kW/m^12^. During these sessions, all participants surfed with each fin condition in a blinded and randomized order for 22 ± 2 min, performing a mean of 7.3 ± 0.9 turns across 2.1 ± 0.1 waves per session. Objective surfing performance data were recorded by a sport-specific tracking device embedded in each surfboard for 227 waves (with 835 turns), on which the participants surfed a total distance of 30.2 km for a total ride time of 4759 s. Table 2 summarizes the key fin performance outcome measures analyzed for 214 waves on which the surfers performed bottom turns and cutbacks (see Supplementary Table S2). The participants’ surfing performances (in terms of speed) when using the 3D-printed fins were similar to those when surfing using the commercial fin. This result was confirmed by analyzing the ratio of the average speed of tracked waves when using the 3DCOM, G1 and G2 fins compared to tracked waves when using the COM fins. The resulting COM speed ratios (Supplementary Table S2) indicated that the level of surfing performance when using 3D printing fins is consistent with that of commercial fins.

**Table 2:** Means ± 95% confidence intervals (CI) of the participants’ surfing performances across the four fin conditions based on the data collected using the sport-specific device embedded in each surfboard.

Fin condition	COM (95% CI), *n* = 56	3DCOM (95% CI), *n* = 44	G1 (95% CI), *n* = 55	G2 (95% CI), *n* = 59	Overall (95% CI) *n* = 214
Total distance (m)	8372	6005	7837	7943	30,157
Total ride time (s)	1288	954	1232	1285	4759
Total tracked waves	60	47	57	63	227
Total tracked turns	224	164	216	231	835
Average distance (m)	146 ± 12	132 ± 16	140 ± 14	134 ± 11	138 ± 6
Average ride time (s)	23 ± 2	21 ± 2	22 ± 2	21 ± 2	22 ± 1
Average turns per wave	4.1 ± 0.5	3.6 ± 0.4	3.9 ± 0.5	3.6 ± 0.4	3.8 ± 0.2
Top speed (m/s)	9.9 ± 0.3 *	9.6 ± 0.3	9.8 ± 0.2	9.6 ± 0.2	9.7 ± 0.1
Average speed (m/s)	6.5 ± 0.2	6.3 ± 0.2	6.4 ± 0.2	6.3 ± 0.2	6.4 ± 0.1
Average cutback duration (s)	0.96 ± 0.06	0.99 ± 0.09	0.94 ± 0.05	0.94 ± 0.06	0.96 ± 0.03
Average bottom turn duration (s)	0.96 ± 0.12	0.93 ± 0.08	0.95 ± 0.07	0.99 ± 0.06	0.96 ± 0.04
Average cutback magnitude (°)	156 ± 7 *	149 ± 6	156 ± 5*	148 ± 4	152 ± 3
Average bottom turn magnitude (°)	101 ± 7 *	90 ± 8	105 ± 10*	100 ± 5	99 ± 4
Average cutback angular velocity (rad/s)	3.1 ± 0.2*	2.9 ± 0.2	3.1 ± 0.2	3.0 ± 0.2	3.0 ± 0.1
Average bottom turn angular velocity (rad/s)	1.9 ± 0.1	1.8 ± 0.2	2.0 ± 0.1	1.8 ± 0.1	1.9 ± 0.1
Average cutback speed (m/s)	6.8 ± 0.2	6.8 ± 0.3	6.6 ± 0.2	6.7 ± 0.2	6.7 ± 0.1
Average bottom turn speed (m/s)	7.5 ± 0.4	7.3 ± 0.5	7.6 ± 0.4	7.0 ± 0.3	7.3 ± 0.2
Average turn flow	0.90 ± 0.04	0.94 ± 0.05	0.88 ± 0.04*	0.95 ± 0.03	0.92 ± 0.02
Average cutback rail angle (°)	76 ± 5	72 ± 6	77 ± 6	74 ± 6	75 ± 3
Average bottom turn rail angle (°)	39 ± 4	38 ± 3	44 ± 7	45 ± 6	42 ± 3
Average cutback pitch angle (°)	45 ± 4	40 ± 4	42 ± 3	42 ± 3	42 ± 2
Average trace power	5.3 ± 0.5*	4.7 ± 0.5	5.2 ± 0.4	4.6 ± 0.4	4.9 ± 0.2
Average power/inertia	6.5 ± 1.4	5.5 ± 1.2	6.0 ± 1.3	6.0 ± 1.2	6.0 ± 0.6

For all statistical comparisons, the reference fin was the 3DCOM design. The magnitude and rail angles indicated yaw and roll angles, respectively. Statistics were calculated on the number of waves (*n* = 214) where participants performed bottom turns and cutbacks.

Significant values are in [bold].

*Indicates a significant difference compared to the 3DCOM fin condition (*p* = 0.05).

### Perceptions of fin performance

We documented each participant’s perceptions of Drive, Feel, Hold, Speed, Stiffness, and Turnability after surfing with each fin condition using a visual analogue scale (VAS; see Supplementary Table S1 and Fig. S1). The mean values for each qualitative descriptor are presented in Table 3.

**Table 3:** Means ± 95% confidence intervals (CI) of the perceptions of fin performance across the four fin conditions based on data from the visual analogue scales.

Fin condition	COM (95% CI) *n* = 35	3DCOM (95% CI) *n* = 35	G1 (95% CI) *n* = 35	G2 (95% CI) *n* = 35
Observed waves	97	91	96	96
Observed turns	430	418	437	405
Drive (mm)	71 ± 7	69 ± 6	76 ± 7	72 ± 8
Feel (mm)	74 ± 8	68 ± 8	**78** ± 7*	70 ± 8
Hold (mm)	72 ± 7	77 ± 6	74 ± 7	71 ± 7
Speed (mm)	74 ± 7	68 ± 6	**80** ± 5^**^**^	74 ± 7
Stiffness (mm)	66 ± 6	70 ± 6	69 ± 8	68 ± 6
Turnability (mm)	78 ± 8	66 ± 6	72.0 ± 7	70 ± 6

Significant values are in [bold].

For all statistical comparisons, the reference fin was the 3DCOM design.

*Indicates a significant difference from the 3DCOM fin condition (*p* < 0.05).

^Indicates a significant difference from the 3DCOM fin condition (*p* < 0.001).

Before evaluating any relationships between the perceptions of fin performance and objective measures of surfing performance, we analyzed the variance inflation factor (VIF) scores for the fin performance outcome measures to ensure an appropriate subset of independent variables were included in the regression analyses. Subsequently, all variables with a VIF score > 5 were removed, leaving only Top Speed (m/s), Number of Turns, Power/Inertia, Bottom Turn and Cutback Rail Angle (°), and Cutback Pitch Angle (°) for use in prediction models.

**Figure 2:**
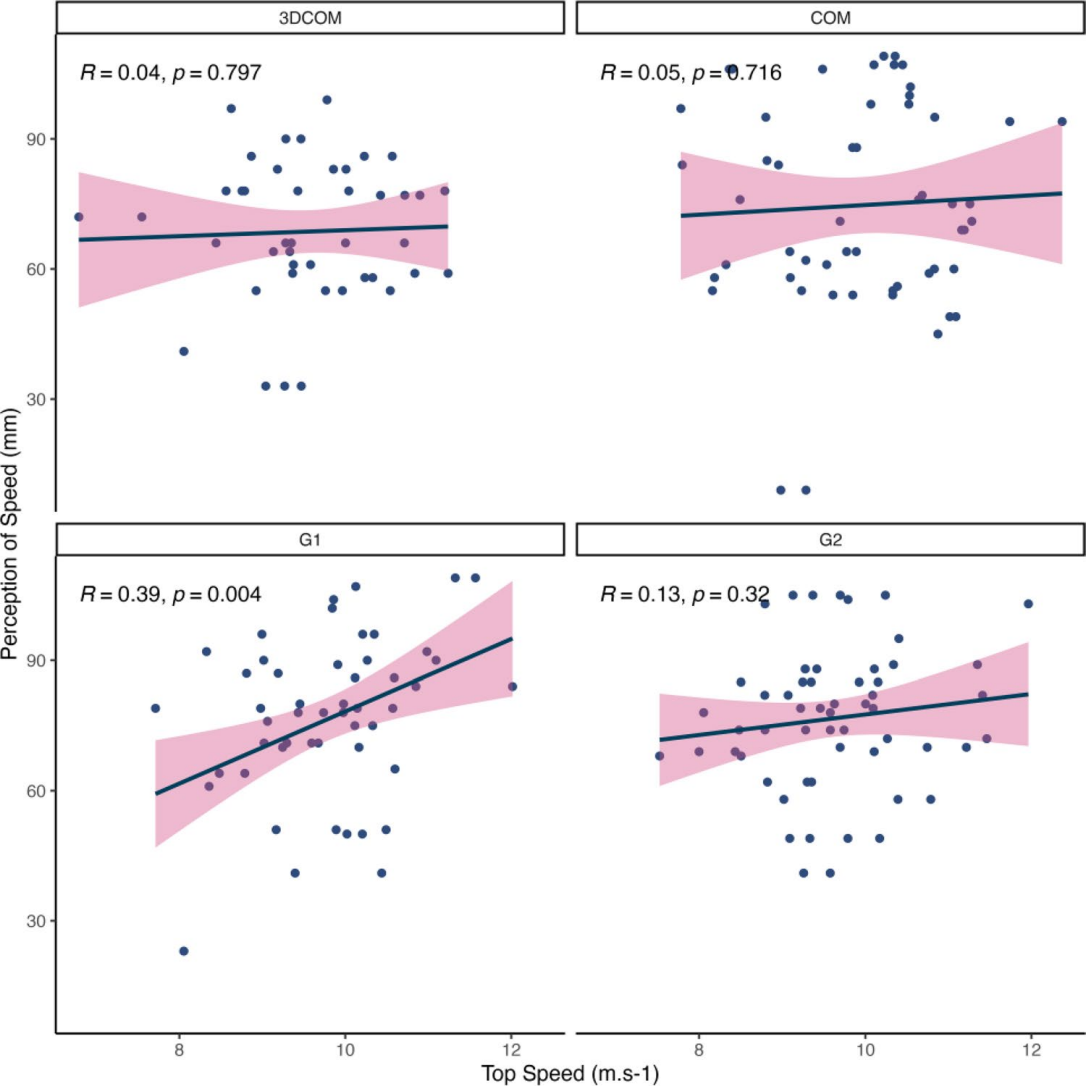
Correlations between the surfers’ perceptions of Speed and their measured Top Speed (m/s), grouped by the fin conditions.

**Figure 3:**
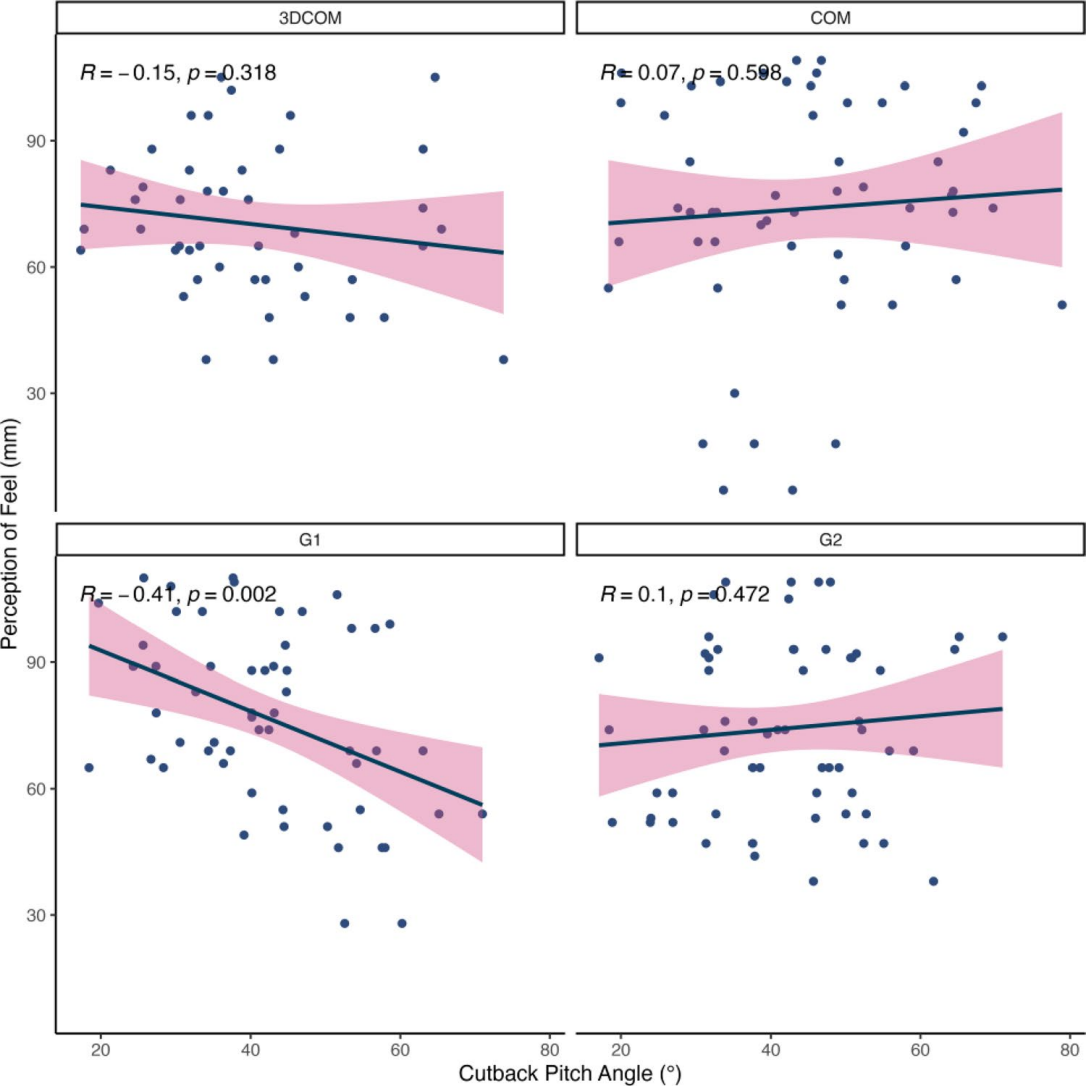
Correlations between the surfers’ perceptions of Feel and their measured Cutback Pitch Angle (°), grouped by the fin conditions.

Visual analogue scale data demonstrating significant differences among the fin conditions (i.e., Speed and Feel) were then correlated with this subset of independent variables to explore possible relationships. From this, significant positive correlations were found for Top Speed and both perceptions of Speed (*r* = 0.39, p = 0.004) and Feel (*r* = 0.29, p = 0.035) in the G1 fin condition (Figs. 2 and S2, respectively). In contrast, negative correlations for the surfers’ perception of Feel were found for Cutback Pitch (*r* = − 0.41, p = 0.002) and rail (*r* = -0.31, p = 0.024) angles in the G1 fin (Figs. 3 and S3, respectively). No other fin condition was correlated with the independent variables.

Optimized combinations of the six independent variables (determined based on second-order Akaike information criterion [AICc] values) were then used to compute linear mixed models of the participants' perceptions of fin performance. That is, we performed six separate analyses to understand the relationships between the perceived fin performance and objective surfing performance. Table 4 summarizes and ranks the performance of the final statistical models. Across the six models produced, five included fixed effects showing a significant relationship between the independent and dependent variables. Three of the five models included the Bottom Turn Rail Angle as a significant predictor of the dependent variable, highlighting its importance in the perception of surfing performance. While these models revealed a significant relationship between the dependent and predictor variables, in general, they did not explain the variance of the dataset well, with conditional *R*^*2*^ values ranging from 0.06 to 0.26. Additional details of each model’s results can be found in the Supplementary Materials (Tables S4–S9).

**Table 4:** Performance metrics for the final six models used to understand the relationship between the perceptions of fin performance (visual analogue scale [VAS] data) and the objective performance recorded by the sport-specific tracking device.

VAS outcome	Conditional R^2^	Marginal R^2^	ICC	RMSE	AICc	AICc weight	Performance score
Drive	0.24	0.03	0.21	13.52	1765.08	0.998	88.6%
Stiffness	0.25	0.02	0.23	17.11	1780.71	0.001	48.4%
Hold	0.26	0.03	0.23	17.61	1793.45	< 0.001	48.0%
Speed	0.11	0.05	0.06	18.16	1801.76	< 0.001	32.2%
Turnability	0.20	0.03	0.17	19.66	1835.49	< 0.001	30.0%
Feel	0.06	0.02	0.05	21.19	1853.36	< 0.001	0.00%

When comparing the performance of the models, maximum likelihood estimations were used as each model did not have the same number of predictors.

ICC, intraclass correlation coefficient; RMSE, root mean square error; AICc, second-order Akaike information criterion.

## Discussion

Additive manufacturing, or 3D printing, is becoming widely used in all industries for rapid prototyping to improve production processes and the quality of the product developed^13,14^. Our results demonstrate that this approach may be a suitable alternative to traditional fin prototyping and production approaches. Most companies that manufacture surfboard fins produce their products through injection molding. This design process requires substantial work to develop and refine the shape before producing the final cast. Here, we have demonstrated that surfboard fins made using 3D printing exhibit comparable structural qualities as well as comparable performance, with no differences found between the COM and any of the 3D printed fins for variables such as mechanical stiffness, top speed, cutback magnitude, and more (see Tables 1 and 2). Previous research^4^ also demonstrated that 3D printing was an acceptable approach to develop and optimize the construction and materials used in 3D printed surfboard fins to have the same mechanical properties as commercial surfboard fins. Therefore, it is plausible that 3D printing of surfboard fins may present an alternative for rapid prototyping and testing of different fin constructions and designs.

In this study, accomplished surfers rode standardized surfboards with fins that differed in how they were constructed (injection mold versus 3D printed) and their design. By using surfboard fins that had been designed to include grooved surface features—in this case, a grooved outer surface (G1) or grooved outer and inner surface (G2)—we were able to evaluate whether an optimized design would contribute to improvements in surfing performance. As previously reported, including a grooved surface on the side fins resulted in an 11% improvement in the lift-to-drag ratio, calculated through computational fluid dynamics (CFD)^2^. This computational process treats fins (and surfboards) as rigid, with a seamless connection between fin and surfboards. In contrast, actual fins and surfboards flex due to the interaction with the water and surfers (i.e., they are not rigid). Moreover, most fins are fitted to a surfboard by inserting fins into fin boxes embedded in the tails of surfboards (i.e., not a seamless connection). This process leads to fins and surfboard dissipating energy through mechanical flex (and vibrations), leading to increased drag and reduced speed. Therefore, the CFD estimations^2^ should be treated as a best-case scenario or the maximum possible improvement in surfing performance.

Interestingly, the data from the tracking device embedded in each participant’s surfboard indicated that grooved fins improved surfing performance. A detailed look at the data collected for the participants over 35 surfing sessions (Table 2 and Supplementary Table S2) revealed that including grooves on the outer edge of a fin improved bottom turn magnitude, cutback turn magnitude, and average turn flow compared to fins without grooves (Table 2). In addition, Supplementary Table S2 (G1 speed ratio) shows that the average speed for most participants (4 out of 6) surfing with single grooved fins was at least 10% faster than when surfing using 3D printed fins without grooves (3DCOM). Overall, the participants’ data showed that their average measured speed was higher on grooved (G1 and G2) fins than 3D printed fins without grooves in 61% of the surfing sessions with viable data.

Including a grooved outer surface reduced the average turn flow for the G1 fins compared to the 3DCOM (Table 2). Although neither the average bottom turn speed nor the cutback speed was significantly different to the 3DCOM, the G1 fin condition allowed the surfers to achieve greater bottom and cutback turn magnitudes, which may have amplified the differences in speed, reducing the average turn flow. In surfing, the bottom turn is used to set the direction and magnitude of the subsequent turn (i.e., cutback)^15^. As the surfer moves from the top of the wave, their acceleration into the apex of the bottom turn is assisted by gravity. Because of this, it is possible that a slight increase in bottom turn speed led to the surfers moving further towards the trough of the breaking wave, allowing a larger magnitude bottom turn to be performed^16^. A larger bottom turn moves the apex of the bottom turn further away from the breaking lip of the wave, thereby allowing the surfer to perform a larger magnitude turn in the critical section of the wave, as evidenced by the larger cutback magnitude (Table 2). Due to this relationship between bottom turn speed and magnitude, our results suggest that due to the greater bottom turn magnitude in the G1 condition, surfers were able to perform cutback turns that were more vertical (relative to the direction of travel along the wave) and had more pronounced changes of direction, thus reducing their average cutback speed. This change in technique is particularly impactful for competitive surfers who would be rewarded for this surfing style^17^. However, given the multifactorial nature of surfing performance, it is difficult to identify whether the fin’s design was responsible for this outcome. We recommend further studies that systematically manipulate surfboard fin characteristics and assess their influence on discrete surfing maneuvers to further support or refute the results of our study.

On average, the participants perceived the G1 fin condition to have better drive, improved turnability, greater speed, and a superior feel when they surfed compared to the 3DCOM fin condition, with the latter two being significantly different (p < 0.05; see Table 3). Furthermore, the G1 fin condition was found to be significantly correlated to multiple measures of surfing performance. For example, top speed and the surfers’ perceptions of speed during each surfing bout with the G1 fin were associated, suggesting that the surfers could reasonably detect when they were surfing faster or slower when using this fin. Interestingly, only the G1 fin condition demonstrated any association with the surfing performance data. We attribute this result to improving the lift-to-drag ratio identified in the CFD analysis of a grooved leading edge in surfboard fins^2^. It is reasonable to suggest that the improved lift-to-drag ratio in the G1 fin condition could have reduced the mechanical vibration of the fins and surfboard as they passed through the moving water on the wave, enhancing the surfers’ lower body proprioception and, in turn, the human-equipment interaction. It is well established that local muscle/tendon vibration can manipulate an individual’s perception of joint position and performance during motor tasks^18–21^. Although it has been found that surfers have much greater postural control and proprioceptive abilities than non-surfers^22,23^, it is not clear whether this results in improved interaction with their equipment and the environment. However, the results of this study may provide some preliminary links to improved perceptions of performance following changes to the design of surfing equipment, similar to that previously found in golf^9^, although further research is needed to replicate these findings.

In addition to the correlations highlighted above, we modelled all the perceived characteristics of the fins through the combinations of the participants' top speed of the wave, number of turns completed, bottom turn and cutback rail angle, and the cutback pitch angle. In particular, the higher performing models of perceived drive, stiffness and hold provide some insights into what measures of surfing performance are related to these qualitative terms. When considering the variables related to perceptions of drive (i.e., the perceived ability to carry speed through turning maneuvers), top speed had a significant, positive relationship. Regardless of the fin condition, a surfer’s objective speed directly influenced their awareness of the fin’s ability to carry speed through maneuvers. Similarly, a fin’s perceived stiffness was inversely related to the surfers’ bottom turn rail angle. In this scenario, participants rated the fins as having greater stiffness when they performed bottom turns with reduced rail angle (i.e., they produced less ‘roll’ of the surfboard along the longitudinal axis). This result may be explained by a perceived difficulty in engaging the inside rail of the surfboard, thereby limiting the rail angle reached during the bottom turn. Being restricted in this way would mean that a surfer’s bottom turn may have been extended, with the apex of the bottom turn progressing too far in front of the trough of the wave, thus reducing the speed carried through the turn. It is worth noting, however, that these mixed models showed high variability (*R*^*2*^ < 0.26) due to the multifactorial and complex nature of surfing in an uncontrolled outdoor environment. As such, more detailed analyses of surfing maneuvers are recommended to evaluate whether this relationship exists in various wave shapes, directions, and conditions. Furthermore, replicating this study in a controlled environment, such as a wave pool, could reduce the models' variability and support the findings we have presented here.

Although this study has demonstrated interesting and unique findings on 3D-printed surfboard fins and our ability to measure surfing performance with high ecological validity, some limitations must be acknowledged. While there are substantial data to support our findings, this study involved six participants (all male). In recruiting participants, we sought to ensure that all participants were experienced and accomplished surfers, were invested in the sport more than just as recreational participants (i.e., a surf coach and surfboard shaper), and that they could accurately evaluate surfing equipment. This level of surfing experience would, therefore, minimize the variability introduced by the novelty of the experiment in their subjective assessments of the fin conditions. A further consideration is that the study participants only surfed in one direction (i.e., a left-hander, a wave that, as the surfer rides the wave, moves to the left as it approaches the shore). However, we do not believe this significantly influenced the study results (due to the balanced number of surfers of each stance). It should also be noted that the sport-specific device collected and calculated surfing performance outcome variables through a proprietary software using 10 Hz GPS data. This is common in the field and sampling rates of 10 Hz have been shown to be reliable and accurate when measuring variables such as peak speed and distance^24–26^. However, this technology is not without its limitations and some authors have suggested that GPS has the potential to underestimate distance, and overestimate velocity, based on the combination of both horizontal and vertical movement of the surfer across a wave face^27^. In addition to this, the device filtered any waves on which turns were performed with a cutback magnitude (yaw angle) smaller than 90 degrees. This led to a difference in the number of observed waves (380) and turns (1690) compared to the tracked waves (227) and turns (835). This is unlikely to impact the study results because each fin condition had a similar representation of measured data across all participants. Furthermore, the participants experienced a range of surfing conditions across the testing period. For example, the swell size was larger at the beginning of the testing period, leading to faster top speeds during wave riding for all individuals. However, all participants could participate in all ranges of conditions, limiting the influence of the conditions on their overall surfing performance and perceptions of surfboard fin performance. To maximise the surfer’s ability perceive differences in the fin conditions, we need studies that better control environmental factors such as wave shape and surfing conditions to understand how changes to fin design influence a surfer’s functional performance (e.g., performing the testing in a wave pool).

In conclusion, we have investigated whether accomplished surfers could accurately perceive how changes to surfboard fin design affected their surfing performance by investigating the relationships between objective data characterizing each surfer’s performance (obtained from a sport-specific tracking device embedded in surfboards) and the surfer’s subjective perceptions while riding waves. The changes to surfboard fin design led to a few objective differences in the fins’ functional performance, with the most substantial changes seen in the improvement in bottom turn and cutback magnitude in the G1 condition (relative to the 3DCOM alternative). The ability to perform larger turns is valuable for competitive surfers, who would be rewarded for performing ‘bigger’ maneuvers, especially if they are completed in the more critical sections of the wave. An optimized fin design with an improved lift-to-drag ratio may therefore give surfers a competitive advantage.

The surfers’ perceptions of how changes to fin design affected their surfing performance mainly agreed with the objective data. Participants could reasonably detect speed changes when surfing using the G1 fin condition. In addition, combinations of the surfers’ top speed when riding waves, bottom turn rail angle, and more were highlighted as having significant relationships with the surfers’ perceptions of the fin’s functional qualities. Although these models are underscored by the high variability associated with measuring surfing performance in the field, they provide insight into the performance qualities that surfers (and subsequently fin manufacturers) can relate to the functions of their fins (e.g., drive, stiffness, and hold). Using a sport-specific tracking device, therefore, presents an exciting opportunity to quantify the functional and performance characteristics of surfboard fins, allowing stakeholders in all areas of the surfing equipment market (e.g., surfers, fin and/or surfboard manufacturers, surfing researchers) more direct information about how surfers can expect their fins to perform. This could increase the possibility for rapid prototyping of fin designs and constructions, leading to faster innovations in the surfing equipment market and better surfing outcomes for all.

## Methods

### Study design

Details of how we developed the 3D-printed fins are published elsewhere^2,4^. In brief, we used a commercially available, medium-size fin template (COM) to inform the design of a 3D-printed alternative made of carbon fiber composite materials (3DCOM), and subsequent experimental iterations of the 3D-printed fins with grooved surface features (G1, G2). These fin designs were then mechanically tested to confirm their similarity to the COM fin before being tested in the ocean by surfers.

We recruited six accomplished surfers from the Illawarra area to participate in the study. Participants were required to comprehensively understand surfing equipment and its role in surfing performance, as determined through their occupation or involvement in the surfing community. As such, the participants included surf retail store managers (2), a surfboard shaper, a surfing coach, a surf club judge, and a surfing science researcher, with an average surfing experience of 22 ± 5 years. There was no a priori sample size estimation as participant numbers were limited by the financial cost as opposed to recruitment possibility. All participants were informed of the aims, procedures, and risks of participating in the study before giving their written, informed consent. This consent included the collection and analysis of performance and video data which would later be used for publication in academic journals. The institution’s Human Research Ethics Committee approved all testing procedures (HE17/174) which were performed in accordance with the Declaration of Helsinki.

Upon study enrolment, participants were invited to an information and testing session where they were provided with detailed information about transportation to the testing location, requirements during the testing window, and how to rate fin performance using the VAS scale. Participants were then transported to a remote surfing location to test the performance of the control and experimental fin conditions. The surfing location was chosen because of its reliable wave shape and length, allowing the participants ample opportunity to test the performance of each fin condition. Over seven days, participants surfed in six sessions, completing four surfing bouts in which they were randomly allocated fin conditions to test. During each surfing bout, surfers had their performance monitored via a sport-specific tracking device embedded in their surfboard (TraceUp, USA). Upon executing approximately 8–10 turning maneuvers, surfers completed a qualitative assessment of the fin’s performance while the research team changed the participant’s fin. We painted every fin black, and each participant was blinded to the fin condition during each surfing bout.

### Fin design and surfing performance

The 3DCOM, G1 and G2 3D-printed fins (on single and double-tab fin bases) were designed using computer-aided design (CAD, Solidworks) based on previous work involving computational fluid dynamics^2^. A commercial 3D printer (Markforged Mark2, USA) was then used to fabricate the fins using a continuous fiber reinforcement method, which lays a continuous carbon fiber during fused filament fabrication of a nylon-carbon composite (ONYX, USA). Commercial single (Futures AM1 Honeycomb Thruster, size medium) and double-tab fins (FCS II Al Merrrick Honeycomb Thruster, size medium) were purchased as the control fin condition.

The mechanical characteristics of the four fin designs were characterized by a custom-built setup consisting of a universal mechanical analyzer (Shimadzu EZ-S, Japan) and a sample holder for clamping fins at the base^4^. This setup was used to evaluate the force required to flex the tip of the fins in the direction perpendicular to the fin using a circular probe (diameter 5 mm) at a rate of 10 mm/min.

All fin sets were fitted to surfboards designed and manufactured by DP Surfboards (Thirroul, NSW, Australia). All surfers used the same surfboard design (DP Surfboards Performa 2.0) with characteristic dimensions of length, width, thickness, volume, and weight are 5′10″, 18 5/8″, 2 ¼′′, 25.75 L and 2.6 kg, respectively. The dimensions of each surfboard were adjusted for each surfer’s height and mass. The surfboards were retrofitted with a custom-designed (using CAD) and 3D printed (on the Markforged) holder to embed the sport-specific tracking devices flush with the deck of the surfboards.

The functional performance of the surfboard fins was measured via a sport-specific tracking device (TraceUp, USA), which fused GPS (10 Hz) and inertial measurement units (200 Hz) to characterize wave-riding performance. This device recorded continuous data during each surfing bout, which was later uploaded to the manufacturer’s server and extracted using a custom-built online data interface. From this, discrete outcome measures for each “turn” (which included both the bottom turn and cutback/top turn executed), such as bottom turn magnitude (º), bottom turn speed (m.s^-1^), and bottom turn rail angle (º), as well as wave summary statistics like ride length (m) and top speed (m.s^-1^) were calculated (see Supplementary Table S10 for an exhaustive list).

During each surfing bout, participants were asked to complete approximately 8–10 turns over 1–2 waves before a researcher changed the participant’s fins for the next surfing bout. Two research team members monitored each surfing bout from a boat in the channel to verify that each participant had completed the required number of turns/waves. Throughout each surfing bout, the number of turns and waves was recorded by the research team (experienced in judging waves and evaluating surfing maneuvers). During our initial examination of the wave-riding data, we removed 13 waves because the ride was too short, or the surfer completed less than one turn. This process resulted in 214 waves with 835 turns for statistical analysis.

### Perceptions of fin performance

At the end of each surfing bout (i.e., following 8–10 completed turns on a fin condition), a researcher presented the participants with six 11-cm VAS scales. Each scale represented common terminology used in surfing to describe the functional characteristics of surfboard fins (Drive, Feel, Hold, Speed, Stiffness, Turnability) and were appropriately anchored at each end. The participants placed a clear mark along the 11-cm scale to indicate how they perceived the fin affected their surfing performance. Two further VAS scales were provided to characterize each surfer’s perception of effort during the surfing bout (see Supplementary Fig. S1).

### Statistical analysis

Means and 95% confidence intervals were computed to characterize the outcome variables of interest. Because of the high number of samples (n = 214), normality was visually assessed for all variables using Q-Q plots because the Shapiro–Wilk test becomes more sensitive to outliers as the sample size increases. Linear mixed models were run using the *lme4* package to assess the differences in the performance outcomes of each fin condition. For all comparisons, two analyses were undertaken—to compare (1) COM to 3DCOM, and (2) to compare the 3DCOM to G1 and G2. For all comparisons, the fin condition (i.e., COM, 3DCOM) was included as the fixed effect and the participant was treated as a random effect. Significant differences in the estimated marginal means were reported. Pearson product-moment correlations were performed to identify associations between perceptions of the functional characteristics of the fins and their objective performance. Correlation coefficients were interpreted as negligible (< 0.30), low (0.3–0.5), moderate (0.5–0.7), large (0.7–0.9), and very large (> 0.9)^28^.

Further linear mixed models were performed to determine whether causal relationships existed between the perception data (VAS scores) and the fin performance outcome variables. Before selecting the most appropriate models, the number of independent variables was reduced through an iterative VIF process that removed all outcome variables that scored > 5 until we found a subset of independent variables. Prediction models for all perception outcome variables (i.e., Drive, Feel, Hold) were then constructed with all possible combinations of the subset of independent variables as the fixed effects, with maximum likelihood estimates used to evaluate the best-performing model for each perception outcome variable (using the AICc metric as the criterion). Once the ideal model was identified, final linear mixed models were computed with restricted maximum likelihood estimates. The participant was used as the random effect for the models generated above. All analyses were performed using RStudio (version 4.3.1; Vienna, Austria) with the *lme4*, *car* and *performance* packages. An alpha level of p < 0.05 was used for all tests.

### Supplementary Information

Supplementary Information.

## Data Availability

Data are not publicly available. However, the analyzed data can be supplied to the corresponding author(s) upon a reasonable request.
