# Supplementary material for: Understanding the relationship between surfing performance and fin design
Source: Sci Rep. 2024 Apr 16;14:8734. doi: 10.1038/s41598-024-58387-y (PMC11021506; doi:10.1038/s41598-024-58387-y)
Supplement: Supplementary file 1 — Supplementary Information. [file 41598_2024_58387_MOESM1_ESM.docx]

Understanding the relationship between surfing performance and fin design

**Supplementary Materials**

**Perceptual Data Definitions**

**Table S1.** Definitions of the six perceptions of surfboard fin performance that the participants judged during surfing bouts.

| **Perceptual Variable** | **Definition** |
| --- | --- |
| *Drive* | Drive relates to the perceived ability of the surfboard and fins to carry or maintain speed throughout a turning manoeuvre. |
| *Feel* | In the context of this study, feel was an overall feeling of the surfboard fin’s performance. |
| *Hold* | Hold is the perceived ability of the surfboard and its fins to maintain a connection to, or grip on, the wave. |
| *Speed* | Speed is the perceived ability to generate and maintain speed when riding along the wave. |
| *Stiffness* | Stiffness relates to the perception of the fin’s (and surfboard’s) response to changes in force production as the surfer manoeuvres the surfboard across the wave. |
| *Turnability* | Turnability relates to the perception of how the surfer can move the surfboard through the water as they complete turning manoeuvres. |

**Surfing Performance Data**

**Table S2.** Means and 95% confidence intervals of the surfing performance across the six participants based on data collected using the sport-specific device embedded into each surfboard. COM speed ratio indicates the ratio of the average speed of tracked waves when using the 3DCOM, G1, and G2 fins compared to tracked waves with the COM fins. G1 and G2 speed ratio indicates the ratio of the average speed of tracked waves when using the G1 and G2 fins compared to the 3DCOM fins, respectively. G1 session and G2 session data represent the percentage of sessions where the average speed of tracked waves when using the G1 and G2 fins was higher than when using the 3DCOM fins, respectively.

|  | **Participant 1** | **Participant 2** | **Participant 3** | **Participant 4** | **Participant 5** | **Participant 6** | **Overall** |
| --- | --- | --- | --- | --- | --- | --- | --- |
| Sessions surfed | 6 | 5 | 6 | 6 | 6 | 6 | 35 |
| Tracked waves | 41 | 29 | 37 | 44 | 31 | 32 | 214 |
| Tracked turns | 167 | 117 | 166 | 181 | 94 | 110 | 814 |
| Total ride time (s) | 880 | 653 | 867 | 939 | 596 | 724 | 4659 |
| Total distance (m) | 5650 | 4308 | 5466 | 5828 | 3866 | 4592 | 29610 |
| COM speed ratio | 1.03 ± 0.04 | 1.1 ± 0.1 | 1.01 ± 0.05 | 0.92 ± 0.05 | 0.91 ± 0.07 | 1.0 ± 0.1 | 0.98 ± 0.03 |
| G1 speed ratio | 1.05 ± 0.06 | 0.92 ± 0.04 | 1.1 ± 0.1 | 1.1 ± 0.1 | 1.1 ± 0.1 | 1.14 ± 0.06 | 1.07 ± 0.06 |
| G1 session (%) | 100 | 0 | 50 | 67 | 67 | 100 | 61 |
| G2 speed ratio | 1.05 ± 0.01 | 0.94 ± 0.05 | 1.09 ± 0.04 | 1.1 ± 0.2 | 1.01 ± 0.05 | 1.15 ± 0.06 | 1.05 ± 0.03 |
| G2 session (%) | 100 | 0 | 100 | 50 | 50 | 100 | 61 |
| COM turns/wave | 4.7 ± 0.4 | 4.0 ± 0.7 | 5.1 ± 0.7 | 3.1 ± 0.7 | 2.7 ± 0.8 | 2.8 ± 0.6 | 3.7 ± 0.1 |
| 3DCOM turns/wave | 3.7 ± 0.4 | 3.4 ± 0.5 | 3.7 ± 0.8 | 3.4 ± 0.8 | 3.0 ± 0.4 | 4.4 ± 0.5 | 3.6 ± 0.1 |
| G1 turns/wave | 2.8 ± 0.6 | 4.9 ± 0.6 | 4.5 ± 0.7 | 3.5 ± 0.7 | 3.3 ± 0.4 | 4 ± 1 | 3.8 ± 0.1 |
| G2 turns/wave | 4.4 ± 0.7 | 3.6 ± 0.7 | 4 ± 1 | 4 ± 1 | 2.9 ± 0.8 | 2.3 ± 0.4 | 3.6 ± 0.1 |

**Table S3.** Means and 95% confidence intervals of the wave-riding performance across the six participants based on data from observation.

|  | **Participant 1** | **Participant 2** | **Participant 3** | **Participant 4** | **Participant 5** | **Participant 6** | **Overall** |
| --- | --- | --- | --- | --- | --- | --- | --- |
| Sessions surfed | 6 | 5 | 6 | 6 | 6 | 6 | 35 |
| Observed waves | 69 | 49 | 68 | 68 | 67 | 59 | 380 |
| Observed turns | 334 | 250 | 336 | 279 | 227 | 264 | 1690 |
| COM turns/wave | 5.4 ± 0.6 | 4.8 ± 0.6 | 5.2 ± 0.3 | 4.1 ± 0.4 | 3.6 ± 0.6 | 4.1 ± 0.4 | 4.5 ± 0.1 |
| 3DCOM turns/wave | 5.2 ± 0.4 | 5 ± 2 | 4.6 ± 0.6 | 4.6 ± 0.5 | 4.5 ± 0.8 | 4.6 ± 0.5 | 4.8 ± 0.1 |
| G1 turns/wave | 4.6 ± 0.3 | 5.5 ± 0.7 | 5.8 ± 0.4 | 4.1 ± 0.4 | 3.3 ± 0.2 | 4.1 ± 0.4 | 4.6 ± 0.1 |
| G2 turns/wave | 4.5 ± 0.4 | 5.2 ± 0.9 | 4.7 ± 0.6 | 3.9 ± 0.5 | 3.1 ± 0.3 | 4.6 ± 0.5 | 4.3 ± 0.1 |

**Full Model Outputs**

**Table S4.** A summary of the model explaining the participant’s perception of Drive. The fixed effects included in the model were based upon the highest performing combination, evaluated on the second order Akaike Information Criteria, as described in the manuscript. The model was fit using restricted maximum likelihood estimation.

|  | **Perception of ‘Drive’** | | | | |
| --- | --- | --- | --- | --- | --- |
| *Predictors* | *Estimates* | *SE* | *95% CI* | *p* | *df* |
| (Intercept) | 51.95 | 13.57 | 25.18 – 78.72 | <**0.001** | 176.44 |
| Top Speed (m.s^-1^) | 2.57 | 1.26 | 0.10 – 5.05 | **0.042** | 199.47 |
| CB Pitch Angle (°) | -0.15 | 0.09 | -0.34 – 0.03 | 0.104 | 200.74 |
| **Random Effects** |  |  |  |  |  |
| σ^2^ | 283.16 |  |  |  |  |
| τ_00 Participant_ | 77.19 |  |  |  |  |
| ICC | 0.21 |  |  |  |  |
| N _Participant_ | 6 |  |  |  |  |
| Observations | 206 |  |  |  |  |
| Marginal R^2^ / Conditional R^2^ | 0.027 / 0.236 | | | | |

*SE: standard error, CI: confidence intervals, CB: cutback, df: degrees of freedom, σ^2^: random effect variance, τ_00_: between participant variance.*

**Table S5.** A summary of the model explaining the participant’s perception of Feel. The fixed effects included in the model were based upon the highest performing combination, evaluated on the second order Akaike Information Criteria, as described in the manuscript. The model was fit using restricted maximum likelihood estimation.

|  | **Perception of ‘Feel’** | | | | |
| --- | --- | --- | --- | --- | --- |
| *Predictors* | *Estimates* | *SE* | *95% CI* | *p* | *df* |
| (Intercept) | 43.96 | 15.69 | 13.02 – 74.91 | <**0.001** | 202.72 |
| Top Speed (m.s^-1^) | 3.05 | 1.60 | -0.09 – 6.20 | 0.057 | 202.74 |
| **Random Effects** |  |  |  |  |  |
| σ^2^ | 460.24 |  |  |  |  |
| τ_00 Participant_ | 22.06 |  |  |  |  |
| ICC | 0.05 |  |  |  |  |
| N _Participant_ | 6 |  |  |  |  |
| Observations | 206 |  |  |  |  |
| Marginal R^2^ / Conditional R^2^ | 0.017 / 0.062 | | | | |

*SE: standard error, CI: confidence intervals, df: degrees of freedom, σ^2^: random effect variance, τ_00_: between participant variance.*

**Table S6.** A summary of the model explaining the participant’s perception of Hold. The fixed effects included in the model were based upon the highest performing combination, evaluated on the second order Akaike Information Criteria, as described in the manuscript. The model was fit using restricted maximum likelihood estimation.

|  | **Perception of ‘Hold’** | | | | |
| --- | --- | --- | --- | --- | --- |
| *Predictors* | *Estimates* | *SE* | *95% CI* | *p* | *df* |
| (Intercept) | 78.12 | 7.12 | 63.66 – 92.57 | <**0.001** | 34.31 |
| Number of Turns | 1.33 | 0.83 | -0.30 – 2.96 | 0.109 | 200.33 |
| CB Rail Angle (°) | -0.15 | 0.07 | -0.28 – -0.02 | **0.023** | 201.29 |
| **Random Effects** |  |  |  |  |  |
| σ^2^ | 322.01 |  |  |  |  |
| τ_00 Participant_ | 98.34 |  |  |  |  |
| ICC | 0.23 |  |  |  |  |
| N _Participant_ | 6 |  |  |  |  |
| Observations | 206 |  |  |  |  |
| Marginal R^2^ / Conditional R^2^ | 0.028 / 0.255 | | | | |

*SE: standard error, CI: confidence intervals, CB: cutback, df: degrees of freedom, σ^2^: random effect variance, τ_00_: between participant variance.*

**Table S7.** A summary of the model explaining the participant’s perception of Speed. The fixed effects included in the model were based upon the highest performing combination, evaluated on the second order Akaike Information Criteria, as described in the manuscript. The model was fit using restricted maximum likelihood estimation.

|  | **Perception of ‘Speed’** | | | | |
| --- | --- | --- | --- | --- | --- |
| *Predictors* | *Estimates* | *SE* | *95% CI* | *p* | *df* |
| (Intercept) | 56.89 | 15.19 | 26.94 – 86.85 | <**0.001** | 199.50 |
| Top Speed (m.s^-1^) | 2.86 | 1.39 | 0.11 – 5.61 | **0.041** | 200.56 |
| BT Rail Angle (°) | -0.15 | 0.08 | -0.30 – 0.00 | 0.058 | 201.46 |
| CB Pitch Angle (°) | -0.11 | 0.10 | -0.31 – 0.09 | 0.283 | 202.00 |
| **Random Effects** |  |  |  |  |  |
| σ^2^ | 341.85 |  |  |  |  |
| τ_00 Participant_ | 20.99 |  |  |  |  |
| ICC | 0.06 |  |  |  |  |
| N _Participant_ | 6 |  |  |  |  |
| Observations | 206 |  |  |  |  |
| Marginal R^2^ / Conditional R^2^ | 0.051 / 0.106 | | | | |

*SE: standard error, CI: confidence intervals, BT: bottom turn, CB: cutback, df: degrees of freedom, σ^2^: random effect variance, τ_00_: between participant variance.*

**Table S8.** A summary of the model explaining the participant’s perception of Stiffness. The fixed effects included in the model were based upon the highest performing combination, evaluated on the second order Akaike Information Criteria, as described in the manuscript. The model was fit using restricted maximum likelihood estimation.

|  | **Perception of ‘Stiffness’** | | | | |
| --- | --- | --- | --- | --- | --- |
| *Predictors* | *Estimates* | *SE* | *95% CI* | *p* | *df* |
| (Intercept) | 73.70 | 4.99 | 62.72 – 84.68 | <**0.001** | 11.10 |
| BT Rail Angle (°) | -0.17 | 0.07 | -0.31 – -0.03 | **0.016** | 200.54 |
| **Random Effects** |  |  |  |  |  |
| σ^2^ | 302.41 |  |  |  |  |
| τ_00 Participant_ | 90.67 |  |  |  |  |
| ICC | 0.23 |  |  |  |  |
| N _Participant_ | 6 |  |  |  |  |
| Observations | 206 |  |  |  |  |
| Marginal R^2^ / Conditional R^2^ | 0.023 / 0.248 | | | | |

*SE: standard error, CI: confidence intervals, CB: cutback, df: degrees of freedom, σ^2^: random effect variance, τ_00_: between participant variance.*

**Table S9.** A summary of the model explaining the participant’s perception of Turnability. The fixed effects included in the model were based upon the highest performing combination, evaluated on the second order Akaike Information Criteria, as described in the manuscript. The model was fit using restricted maximum likelihood estimation.

|  | **Perception of ‘Turnability’** | | | | |
| --- | --- | --- | --- | --- | --- |
| *Predictors* | *Estimates* | *SE* | *95% CI* | *p* | *df* |
| (Intercept) | 63.52 | 5.17 | 52.43 – 74.61 | <**0.001** | 199.50 |
| BT Rail Angle (°) | 0.22 | 0.08 | 0.06 – 0.38 | **0.007** | 201.46 |
| **Random Effects** |  |  |  |  |  |
| σ^2^ | 398.76 |  |  |  |  |
| τ_00 Participant_ | 82.75 |  |  |  |  |
| ICC | 0.17 |  |  |  |  |
| N _Participant_ | 6 |  |  |  |  |
| Observations | 206 |  |  |  |  |
| Marginal R^2^ / Conditional R^2^ | 0.030 / 0.197 | | | | |

*SE: standard error, CI: confidence intervals, CB: cutback, df: degrees of freedom, σ^2^: random effect variance, τ_00_: between participant variance.*

**Surf-specific Tracking Device Variables**

**Table S10.** A summary of the outcome variables produced from the sport-specific surfboard tracker. These variables are calculated through proprietary algorithms based on GPS, accelerometer, gyroscope and magnetometer data.

| **Outcome Variable** | **Definition** |
| --- | --- |
| Average Speed (m.s^-1^) | The average speed when riding a wave. |
| Maximum Speed (m.s^-1^) | The peak speed attained when riding a wave. |
| Ride Time (s) | The time elapsed from the take-off (>7 m.s^-1^) and completion of the wave (<7 m.s^-1^) |
| Total Distance (m) | The total distance covered while riding the wave. |
| Number of Turns | The number of completed turning manoeuvres. A completed turn includes a cutback and subsequent bottom turn for this device. |
| Bottom Turn Magnitude (°) | The surfboard’s yaw angle displacement along the inferior surface of the wave (see Gately et al. [4] for more detail). |
| Bottom Turn Speed (m.s^-1^) | The average/peak speed attained during the bottom turn (i.e., the turn performed to redirect the surfer from the inferior surface to the superior surface of the wave’s face). |
| Bottom Turn Duration (s) | The time elapsed during the bottom turn. |
| Bottom Turn Angular Velocity (rad.s^-1^) | The bottom turn angular displacement over the time elapsed to complete the bottom turn. |
| Bottom Turn Rail Angle (°) | The surfboard’s roll angle displacement along the inferior surface of the wave (see Gately et al. [4] for more detail). |
| Bottom Turn Pitch Angle (°) | The surfboard’s pitch or vertical angle displacement along the inferior surface of the wave (see Gately et al. [4] for more detail). |
| Cutback Magnitude (°) | The surfboard’s yaw angle displacement along the superior surface of the wave (see Gately et al. [4] for more detail). |
| Cutback Speed (m.s^-1^) | The average/peak speed attained during the cutback turn (i.e., turn performed to redirect the surfer from the superior surface to the inferior surface of the wave’s face). |
| Cutback Duration (s) | The time elapsed during the cutback turn. |
| Cutback Angular Velocity (rad.s^-1^) | The cutback turn angular displacement over the time elapsed to complete the cutback turn. |
| Cutback Rail Angle (°) | The surfboard’s roll angle displacement along the superior surface of the wave (see Gately et al. [4] for more detail). |
| Cutback Pitch Angle (°) | The surfboard’s pitch or vertical angle displacement along the superior surface of the wave (see Gately et al. [4] for more detail). |
| Bottom Turn Speed Gain (m.s^-1^) | The difference between the cutback speed and subsequent bottom turn speed attained. |
| Power/Inertia | A measure of total turning power as it eliminates the effects of inertia. It is calculated using the difference in angular velocity between the bottom turn and the cutback turn. |

**Perceptual Data Scale**

**
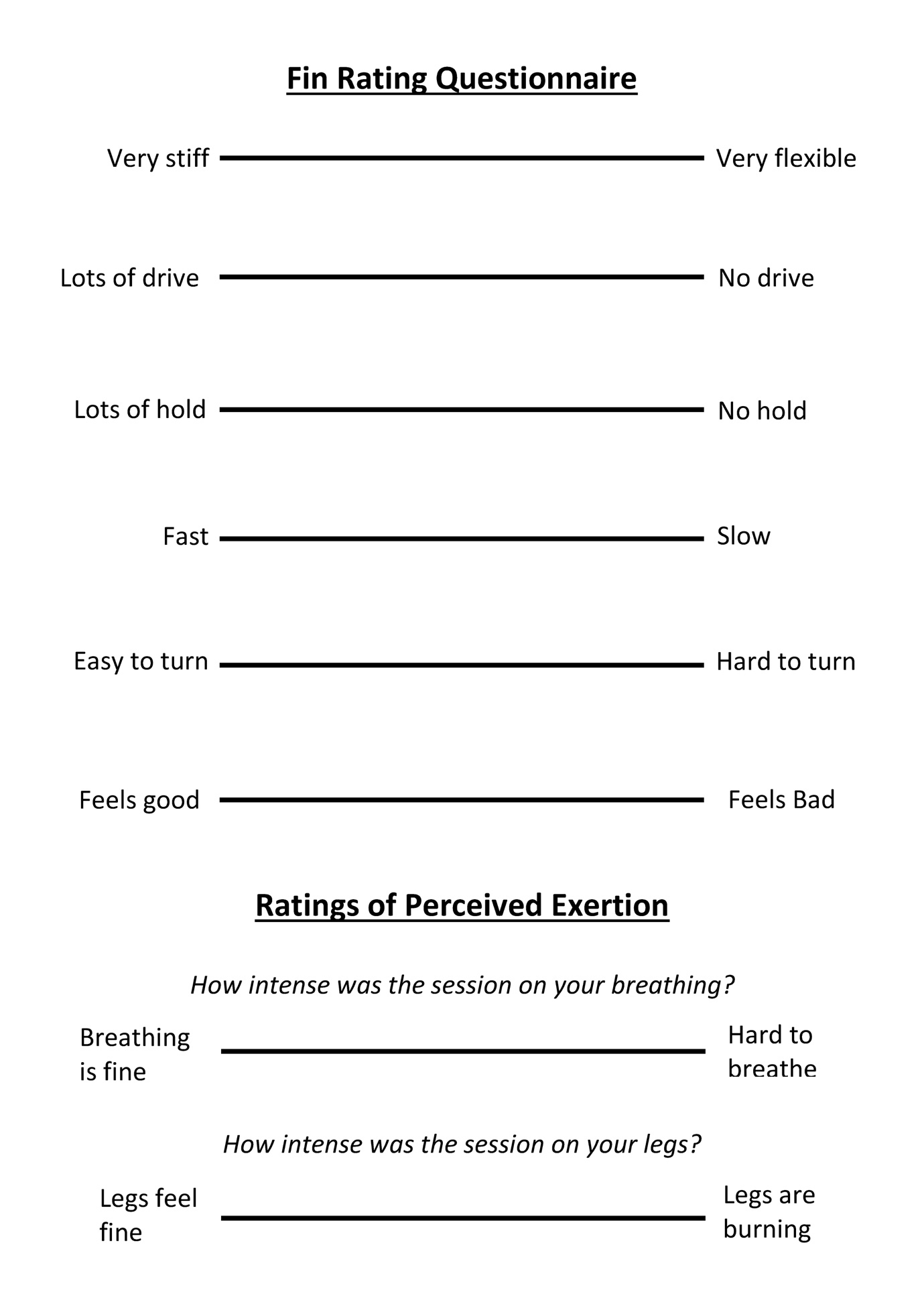
**

**Figure S1.** The Visual Analogue Scale (VAS) used to quantify the participant’s perceptions of the functional performance of the commercial and 3D printed fins. All scales were 11 cm in length and appropriately anchored.

**Fin-level Surfing Performance Correlations**


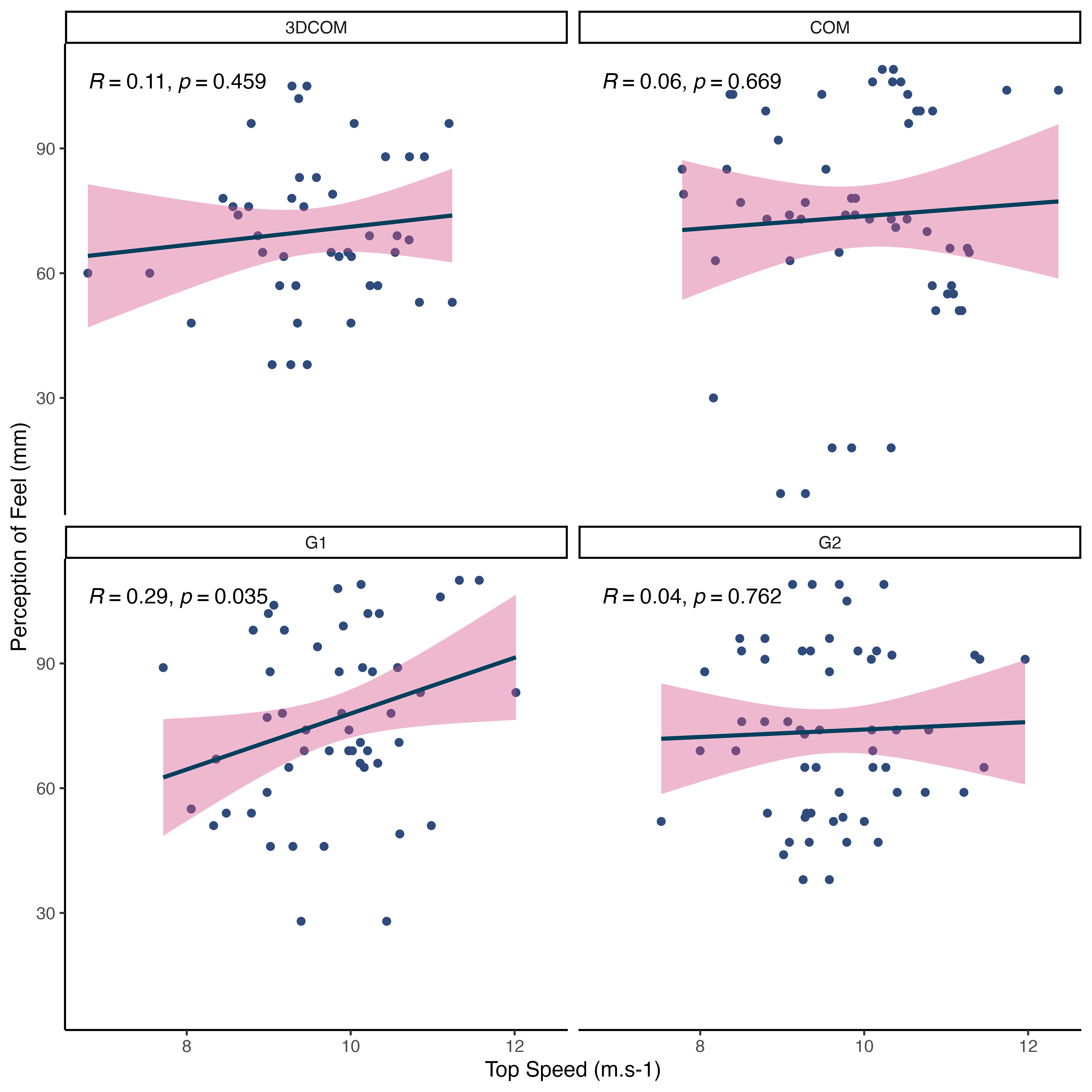


**Figure S2.** Correlations between the surfer’s perceptions of Feel and their measured Top Speed (m.s^-1^), grouped by fin conditions.


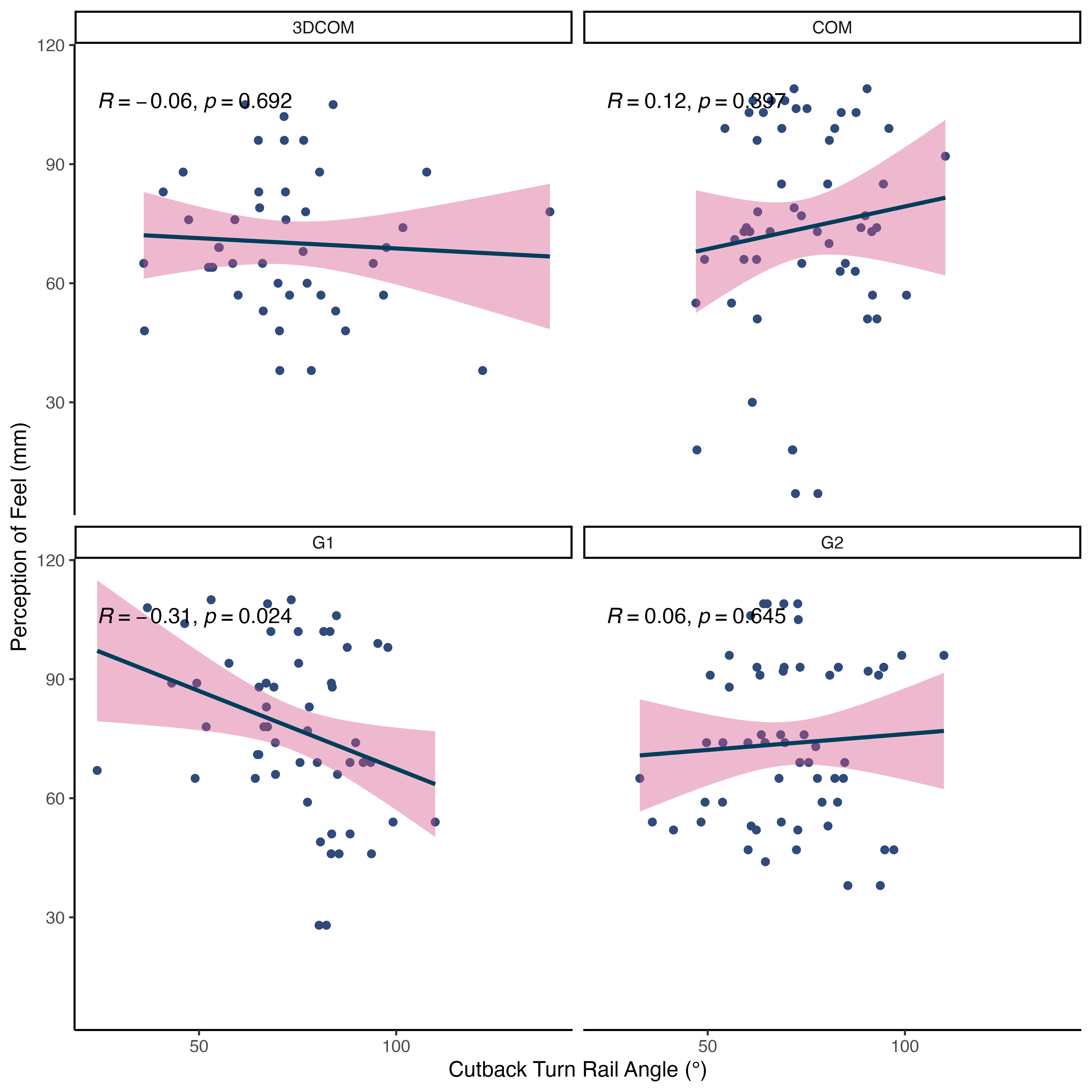


**Figure S3.** Correlations between the surfer’s perceptions of Feel and their measured Cutback Rail Angle (º), grouped by fin conditions.
